# Knockout of Pannexin-1 Induces Hearing Loss

**DOI:** 10.3390/ijms19051332

**Published:** 2018-04-30

**Authors:** Jin Chen, Chun Liang, Liang Zong, Yan Zhu, Hong-Bo Zhao

**Affiliations:** Department of Otolaryngology, University of Kentucky Medical Center, 800 Rose Street, Lexington, KY 40536, USA; catkin19832002@163.com (J.C.); chunliang13@yeah.net (C.L.); cell-099@163.com (L.Z.); yan.zhu@uky.edu (Y.Z.)

**Keywords:** Panx1, deafness, hearing, gap junction, inner ear, ABR, DPOAE, CM

## Abstract

Mutations of gap junction connexin genes induce a high incidence of nonsyndromic hearing loss. Pannexin genes also encode gap junctional proteins in vertebrates. Recent studies demonstrated that Pannexin-1 (Panx1) deficiency in mice and mutation in humans are also associated with hearing loss. So far, several Panx1 knockout (KO) mouse lines were established. In general, these Panx1 KO mouse lines demonstrate consistent phenotypes in most aspects, including hearing loss. However, a recent study reported that a Panx1 KO mouse line, which was created by Genentech Inc., had no hearing loss as measured by the auditory brainstem response (ABR) threshold at low-frequency range (<24 kHz). Here, we used multiple auditory function tests and re-examined hearing function in the Genentech Panx1 (Gen-Panx1) KO mouse. We found that ABR thresholds in the Gen-Panx1 KO mouse were significantly increased, in particular, in the high-frequency region. Moreover, consistent with the increase in ABR threshold, distortion product otoacoustic emission (DPOAE) and cochlear microphonics (CM), which reflect active cochlear amplification and auditory receptor current, respectively, were significantly reduced. These data demonstrated that the Gen-Panx1 KO mouse has hearing loss and further confirmed that Panx1 deficiency can cause deafness.

## 1. Introduction

Gap junctions play a critical role in hearing. It has been found that mutations in the Connexin26 (Cx26, *GJB2*) gap junctional gene induce a high incidence of hearing loss, accounting for more than 50% of the cases of nonsyndromic hearing loss [1,2,3,4,5]. The pannexin gene family also encodes gap junctional proteins in vertebrates. So far, three pannexin isoforms (Panx1, 2, and 3) have been cloned from the human and mouse genomes [6,7]. Like connexins, pannexins have ubiquitous expression in almost all tissues. In the mammalian inner ear, pannexins also have extensive expression; all three pannexin isoforms have expression in the inner ear [8]. Panx1 predominantly expresses at the supporting cells in the organ of Corti, the interdental cells in the spiral limbus, inner and outer sulcus cells, and fibrocytes in the cochlear lateral wall. Panx2 predominantly expresses at the basal cells in the stria vascularis, and Panx3 mainly expresses at the bony structure of the cochlea. However, auditory sensory hair cells have no expression of pannexins [8]. These distinctive cellular distributions strongly suggest that pannexins may have important functions in hearing.

Unlike connexins that form intercellular gap junctional channels, pannexins usually function as undocked gap junctional channels on the plasma membrane to provide an intracellular-extracellular conduit. Due to the relatively large pore size, Panx1 channels in many organs and tissues act as conduits that allow small molecules, such as ATP [9,10,11], to pass through in order to participate in many physiological functions and pathological processes [12,13,14,15,16,17,18,19,20]. Our previous study also demonstrated that Panx1 channels dominate ATP release in the cochlea [20,21]. ATP in the cochlea can mediate hair cells’ sound transduction and neurotransmission [22], outer hair cell (OHC) electromotility [23,24], hearing dynamic range [25], synchronization of auditory nerve activity during development [26,27], gap junctional coupling [28], K^+^-sinking [29], and endocochlear potential (EP) generation [21].

It is well-known that mutations in connexin genes can induce hearing loss [1,2,3,4,5]. However, it was for a long-time undetermined whether Panx deficiency can induce hearing loss, until recent studies showing that Panx1-deficient mice [21,30] and mutation in humans [31] are associated with deafness. So far, several Panx1 knockout (KO) mouse lines have been established [15,21,30,32,33]. In general, these Panx1 KO mice demonstrated consistent phenotypes. However, one Panx1 KO mouse line, which was created by Genentech Inc. (South San Francisco, CA, USA) [15], was reported to have no hearing loss, as measured by auditory brainstem response (ABR) recording at low-frequency range (<24 kHz) [34]. In this study, we re-examined hearing function of this Genentech Panx1 (Gen-Panx1) KO mouse line using multiple auditory functional tests. We found that the Gen-Panx1 KO mice have hearing loss, particularly in the high-frequency region, which is consistent with hearing loss observed in Foxg1-Panx1 conditional knockout (cKO) mice (a line which was created by crossing with a Foxg1-Cre line) [21], and in Pax2-Panx1 cKO mice (a line which was created by crossing with a Pax2-Cre mouse line) [30]. These new data further confirmed that Panx1 deficiency can induce hearing loss.

## 2. Results

### 2.1. Panx1 Deletion in the Cochlea in Gen-Panx1 KO Mice

**Figure 1:**
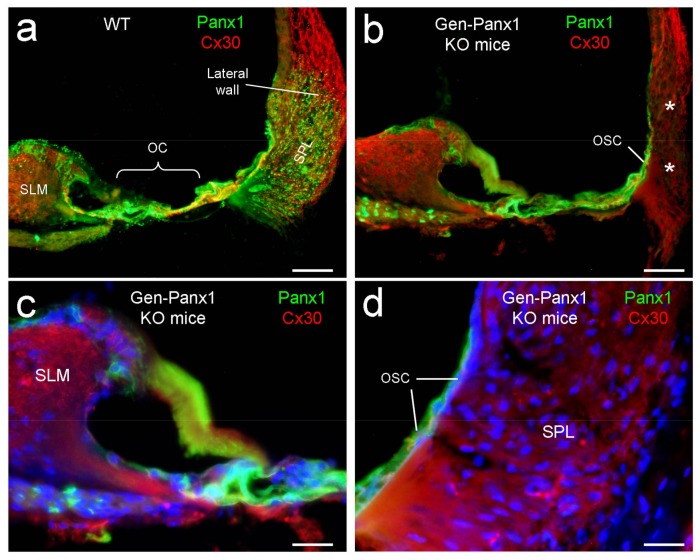
Deletion of Panx1 in the cochlea of Gen-Panx1 knockout (KO) mice. (**a**): Double immunofluorescent staining for Panx1 (green) and Cx30 (red) in the wild-type (WT) mouse cochlea. (**b**–**d**): Panx1 expression and deletion in the cochlea of Gen-Panx1 KO mice. White asterisks in panel (**b**) indicate the absence of Panx1 labeling at the spiral ligament (SPL) in the lateral wall of the Gen-Panx1 KO mice. OC: organ of Corti; OSC: outer sulcus cell; SLM: spiral limbus. Scale bars: (**a**,**b**): 50 µm, (**c**,**d**): 25 µm.

As previously reported [8], Panx1 had predominant expression in the cochlea, including the organ of Corti and the lateral wall (Figure 1a). In the Gen-Panx1 KO mouse, Panx1 expression at the cochlear lateral wall was deleted (Figure 1b,d); Panx1 labeling was completely absent at the spiral ligament (SPL). However, Panx1 labeling at the organ of Corti (OC), the spiral limbus (SLM), and outer sulcus cells (OSCs) remained intense (Figure 1b–d). This deletion pattern in the Gen-Panx1 KO mice is similar to Panx1 deletion in the cochlea in the Foxg1-Panx1 cKO mice [21]. This location- and cell-specific knockout pattern also provided direct evidence for the specificity of the anti-Panx1 antibody used in this study.

Co-labeling for Cx30 showed that there was no apparent difference between Gen-Panx1 KO mice and wild-type (WT) mice (Figure 1). Cx26 expression in the cochlea also appeared normal in the Gen-Panx1 KO mice (data not shown).

### 2.2. Hearing Loss in Gen-Panx1 KO Mice

**Figure 2:**
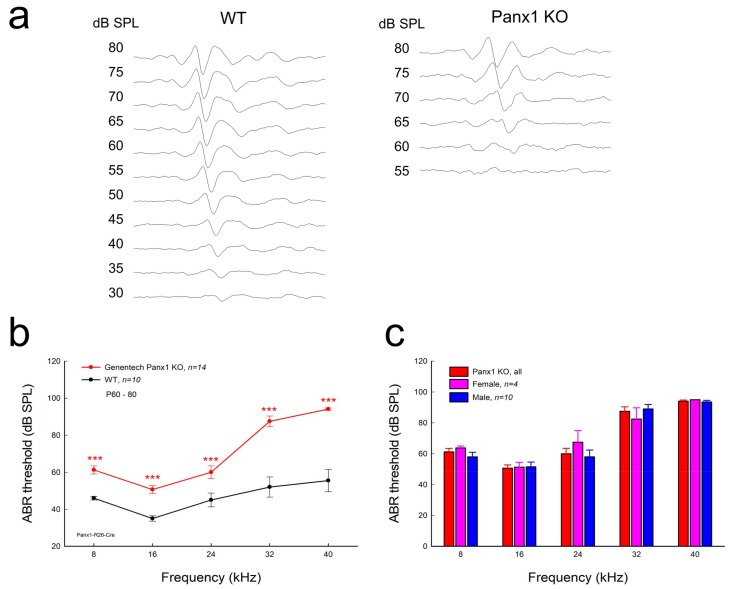
Hearing loss in Gen-Panx1 KO mice. (**a**): auditory brainstem responses (ABRs) in Gen-Panx1 KO and WT mice. The ABR was evoked by a 16 kHz tone burst. (**b**): ABR thresholds measured in Gen-Panx1 KO and WT mice. ABR thresholds in Gen-Panx1 KO mice were increased. *** *p* < 0.001 as determined by one-way ANOVA with a Bonferroni correction. (**c**): There is no significant difference in ABR thresholds between different genders in the Gen-Panx1 KO mice.

Figure 2a-c shows ABR recording in the Gen-Panx1 KO mice. ABR thresholds at 8, 16, 24, 32, and 40 kHz were 60.0 ± 2.28, 50.0 ± 2.28, 57.1 ± 3.30, 86.4 ± 3.21, and 93.9 ± 0.77 dB SPL, respectively (Figure 2b). In comparison with WT mice, ABR thresholds at 8, 16, 24, 32, and 40 kHz in the Gen-Panx1 KO mice were significantly increased by 14.0 ± 2.88, 15.5 ± 2.88, 12.1 ± 3.30, 34.4 ± 3.21, and 38.4 ± 1.77 dB SPL, respectively (*p* < 0.001, one-way ANOVA with a Bonferroni correction). In the high-frequency region, ABR thresholds had larger increases in comparison with those in the low-frequency region (Figure 2b). However, there was no significant difference in ABR thresholds between male and female Gen-Panx1 KO mice (Figure 2c). Thus, we did not separate different genders in data analyses in the following experiments.

### 2.3. Reduction of Distortion Product Otoacoustic Emission in Gen-Panx1 KO Mice

**Figure 3:**
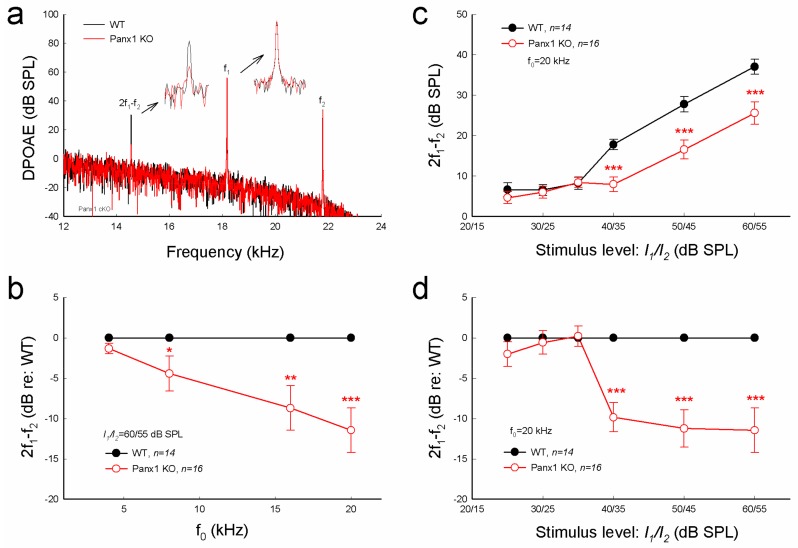
Distortion product otoacoustic emission (DPOAE) reduction in Gen-Panx1 KO mice. (**a**): Evoked spectra of acoustic emission in Panx1 KO mice and in WT mice. Inset: High-magnification plot of 2*f*_1_ − *f*_2_ and *f*_1_ peaks. (**b**): Reduction of DPOAE in frequency responses in the Panx1 KO mice. Magnitudes of 2*f*_1_ − *f*_2_ in the Panx1 KO mice ware normalized to those in WT mice. (**c**,**d**): I-O function of DPOAE in Panx1 KO and WT mice. DPOAEs in panel (**d**) were normalized to those in WT mice. * *p* < 0.05, ** *p* < 0.01, and *** *p* < 0.001 as determined by one-way ANOVA with a Bonferroni correction.

Consistent with increases in ABR threshold (Figure 2), distortion product otoacoustic emission (DPOAE) in the Gen-Panx1 KO mouse was significantly reduced (Figure 3a-d). DPOAEs at *f*_0_ of 20 kHz in the Gen-Panx1 KO mice and WT mice were 25.6 ± 2.76 and 37.1 ± 1.83 dB SPL, respectively (Figure 3c). In comparison with WT mice, DPOAEs in the Gen-Panx1 KO mice at *f*_0_ = 4, 8, 16, and 20 kHz were reduced by −1.32 ± 0.63, −4.43 ± 2.18, −8.71 ± 2.76, and −11.5 ± 2.73 dB, respectively (Figure 3b). As shown in our previous publication [30], the reduction was increased as sound intensity was increased (Figure 3c,d). In I-O plot (Figure 3c), DPOAEs in the Gen-Panx1 KO mice at the stimulus intensity range from 40 to 60 dB SPL were significantly reduced. In comparison with WT mice, the reduction was ≥10 dB SPL (*p* < 0.001, one-way ANOVA with a Bonferroni correction) (Figure 3d). However, at the stimulus intensity lower than 40 dB SPL, DPOAEs in the Gen-Panx1 KO mice and WT mice were not apparently different (Figure 3c,d).

### 2.4. Reduction of Auditory Receptor Potential in Gen-Panx1 KO Mice

**Figure 4:**
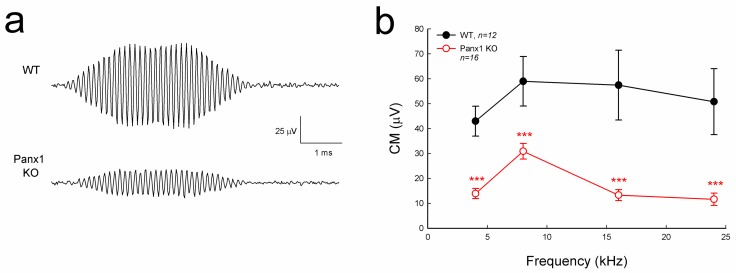
Cochlear microphonics (CM) reduction in the Gen-Panx1 KO mouse. (**a**): CM waveforms recorded from Gen-Panx1 KO and WT mice. (**b**): Reduction of CM in the Panx1 KO mouse. *** *p* < 0.001 as determined by one-way ANOVA with a Bonferroni correction.

Cochlear microphonics (CM) is the auditory receptor current/potential. Figure 4 shows that the CM in the Gen-Panx1 KO mice was reduced. CM in WT mice was 40–60 µV, while CM in the Gen-Panx1 KO mice was 10–30 µV, thus showing a reduction by more than 50% (*p* < 0.001, one-way ANOVA with a Bonferroni correction).

### 2.5. No Apparent Hair Cell Loss in Gen-Panx1 KO Mice

**Figure 5:**
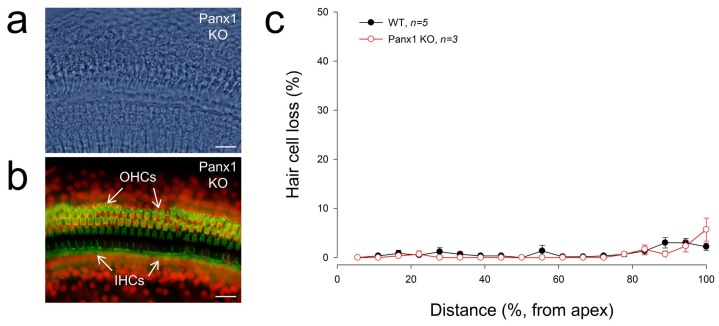
No apparent hair cells in Gen-Panx1 KO mice. (**a**,**b**): The cochlear sensory epithelia of the Gen-Panx1 KO mice in whole-mounting preparation with staining of phalloidin-Alexa Fluor-488 (green) and propidium iodide (red). OHCs: outer hair cells; IHCs: inner hair cells. Scale bars: 20 µm. (**c**): There is no apparent hair cell loss in the Gen-Panx1 KO mice.

Figure 5 shows that there was no substantial hair cell loss in Gen-Panx1 KO mice. The loss of hair cells in the Gen-Panx1 KO mice was less than 5%. There was no significant difference of hair cell loss between WT and Gen-Panx1 KO mice. This is consistent with the previous report that there is no significant hair cell loss in Foxg1-Panx1 cKO mice [21].

## 3. Discussion

In this study, we found that the Gen-Panx1 KO mouse had hearing loss. ABR thresholds were significantly increased, and DPOAE and CM were significantly reduced (Figure 2, Figure 3 and Figure 4). These data are consistent with previous findings that Panx1-deficient mice have hearing loss [21,30] and are also consistent with the fact that Panx1 mutation in humans can cause deafness [31].

In this experiment, in addition to the increases in ABR threshold, we found that DPOAE in the Gen-Panx1 KO mice was reduced (Figure 3). This finding is consistent with our previous findings of DPOAE reduction and hearing loss in Pax2-Panx1 cKO mice [30]. DPOAE reflects the activity of active cochlear amplification, which can increase hearing sensitivity and is required for normal hearing. DPOAE reduction suggests that active cochlear amplification is impaired, which can induce hearing loss and increase ABR threshold (Figure 2). Moreover, in line with the observed reduction of CM in the Foxg1-Panx1 cKO mice [21], CM in this Gen-Panx1 KO mouse line was also reduced (Figure 4). CM is the auditory receptor current/potential. CM reduction can consequently decrease active cochlear amplification, thereby eventually leading to hearing loss. Thus, consistent with ABR recordings, these auditory function tests also demonstrated hearing loss in the Gen-Panx1 KO mice. 

However, these findings are inconsistent with a recent report that the Gen-Panx1 KO mice had no hearing loss in ABR recordings [34]. Several factors could contribute to this discrepancy. First, ABR in that report [34] was recorded at frequency range <24 kHz. However, as shown in Figure 2 and in our previous reports [21,30], the Panx1 deficiency-induced hearing loss was most severe at high-frequency (>24 kHz). By recording at these low frequencies, hearing loss at higher frequencies may have been missed by the authors. Second, the study used C57BL/6 mice rather than WT littermates as control. Although the Gen-Panx1 KO mice have similar genetic backgrounds with C57BL/6 mice, this still could produce significant differences in hearing functional tests. In particular, hearing loss in the Gen-Panx1 KO mice in the middle- and low-frequency range is not as large as that in the high-frequency range (Figure 2b). Third, unlike in the previous report [34], which only considered ABR recordings, in the present study, we also recorded CM and DPOAE (Figure 3 and Figure 4). All of these auditory functional tests demonstrated hearing loss in the Gen-Panx1 KO mouse.

In the previous report [34], the susceptibility of the Gen-Panx1 KO mouse to noise was also examined. Mice were exposed to a high intensity tone (12 kHz, 115 dB SPL, 1 h), and there was no significant difference in ABR thresholds between the Panx1-deficient mice and WT mice measured at the post-exposure day 7 [34]. However, since both WT and KO mice had large ABR threshold shifts for this high-intensity exposure, the susceptibility of Panx1 KO mice to noise could not be assessed. In order to assess susceptibility, low- or moderate-intensity noise exposure could be used to test whether the Panx1-deficient mice have larger threshold shifts than WT mice. In addition, only recording ABR thresholds at the seventh day after noise exposure [34] may have been too short a time to assess a permanent threshold shift (PTS).

Recently, another Panx1 KO mouse line (B6;129-*Panx1*^tm1.Fam/Cnrm^, European Mouse Mutant Archive (EMMA): E11476) [32] has also been reported to have no hearing loss [35]. However, it has been reported that the brain tissues of this EMMA Panx1 KO mouse line showed no negative reaction to Panx1 in Western blotting using several anti-Panx1 antibodies [36], including a chicken anti-human Panx1 antibody used in this study, whose specificity was widely validated in previous experiments by multiple assays in different Panx1 KO mouse lines [8,9,21,30,37,38]. This suggests that this EMMA Panx1 KO mouse line may have a hypomorphic phenotype. As shown in our immunofluorescent staining in this study (Figure 1) and also in previous studies in other Panx1 KO mouse lines [21,30], the location- and cell-specific deletion of Panx1 is clearly visible in the cochlea. This cell-specific deletion pattern also provides direct and unequivocal evidence that the used chicken anti-human antibody is specific to Panx1. Indeed, a hypomorphic phenotype has been found in the KOMP (Knockout Mouse Project, University of California, Davis, CA, USA) Panx1 KO mouse line, in which Panx1 is not completely deleted [33]. Finally, Panx1 deficiency-induced hearing loss is progressive [20,30]; the increase in ABR threshold is small at postnatal day 30 (P30) and becomes large and apparent after P60 [20,30]. The ABR threshold in the previous report [35] was averaged from P30 to P90. This could also attenuate the potential difference. Thus, as suggested by these inconsistent results and reports, Panx1 function including characterization of Panx1 KO mice and the specificity of anti-Panx1 antibodies still needs to receive further study in the future. 

Our previous study demonstrated that the Panx1 deficiency reduced ATP release and EP generation in the cochlea [21]. Positive EP in the cochlear endolymph is generated in the cochlear lateral wall [21,39] and a driving force for K^+^-ions passing through the mechano-transduction channels in hair cells to produce CM [39], although hair cells have neither connexin [40,41] nor pannexin expression [8]. EP reduction can reduce CM, thereby leading to the reduction of active cochlear amplification and eventually hearing loss. In this study, we did not measure EP and ATP release in the Gen-Panx1 KO mice. However, CM and DPOAE were reduced in the Gen-Panx1 KO mice (Figure 3 and Figure 4). Thus, the Gen-Panx1 KO mice may share the same mechanism to reduce CM and active cochlear amplification, thereby leading to hearing loss. 

## 4. Materials and Methods

### 4.1. Panx1 KO Mice and Genotyping

Gen-Panx1 KO mice were created by Vishva Dixit at Genentech Inc. with the loxP-Cre technique [15]. In this transgenic mouse strain, Exon2 of Panx1 was floxed with FLP recombinase and was deleted by crossing to the C57BL/6-Gt(ROSA)26^Sortm16(Cre)^ Arte Cre deleter strain (Taconic Artemis, Rensselaer, NY, USA). Mice were genotyped by tail genomic DNA with PCR primers: 5′-TGA CCA CAG ACA GCA CTTAAG-3′ and 5′-CGT CTG AGA GCT CCC TGG CG-3′, which yield a 651-bp WT band and a 335-bp knockout band [15]. WT littermates served as control. All recordings were performed under anesthesia with Ketamine and Xylazine (8.5 mL saline + 1 mL (100 mg/mL) Ketamine + 0.55 mL (20 mg/mL) Xylazine, 0.1 mL/10 g body weight, ip) to minimize suffering. The experimental procedures were approved by the University of Kentucky’s Animal Care and Use Committee (Protocol No. 00902M2005, May 9, 2010) and conducted according to the standards of the NIH Guidelines for the Care and Use of Laboratory Animals.

### 4.2. Data Processing and Statistical Analysis

A total of 16 Gen-Panx1 KO mice (4 females and 12 males) were used in this study. Littermate WT (6 females and 8 males) mice were used as control. Mouse ages were between P60 to P90. For ABR, DPOAE, and CM recordings, 14 (4 females and 10 males), 16 (4 females and 12 males), and 16 (4 females and 12 males) of Gen-Panx1 KO mice were used, respectively. Exact numbers of mice used in each experiment were also indicated in the corresponding figures. Since there was no significant difference in ABR threshold between different genders in Gen-Panx1 KO mice (Figure 2c), we did not do gender analyses in other experiments. 

The statistical analyses were performed by SPSS v18.0 (SPSS Inc., Chicago, IL, USA) using one-way ANOVA with a Bonferroni correction. The level of statistical significance was set at *p* < 0.05. Data were presented as mean ± SEM and plotted by SigmaPlot v10 (SPSS Inc., Chicago, IL, USA). All sample size and P values were reported in the figures or the figure legends. 

### 4.3. ABR, CM, and DPOAE Recordings

As described in our previous publications [21,42,43,44,45,46], ABR was recorded by a Tucker-Davis ABR & DPOAE workstation with ES-1 high frequency speakers (Tucker-Davis Tech., Alachua, FL, USA). Mouse body temperature was maintained at 37–38 °C. ABR was measured by clicks (rate: 20/s) in alternative polarity and series tone pips (5 ms duration with 1 ms up and down ramp, rate: 20/s) from 80 to 10 dB SPL in a 5-dB step at 8, 16, 24, 32, and 40 kHz. The signal was amplified (50,000×), filtered (300–3000 Hz), and averaged by 500 times. The lowest level at which the ABR could be recognized was defined as the threshold. The high levels of acoustic stimuli (100 to 70 dB SPL) were used if the threshold was >75 dB SPL.

CM was recorded with the same electrode configuration as the ABR recording, i.e., one electrode was inserted at the vertex, one electrode was ventrolaterally inserted to the right or left ear, and the ground needle electrode was inserted in the right leg [42,46]. However, the signal evoked by tone bursts was amplified (50,000×), filtered (3–50 kHz), and averaged by 250 times, as described in our previous publications [42,46].

For DPOAE recording, an ear-plug that contained a small microphone and two small tubes was plugged into the outer ear cannel. Two-testing sounds were separately induced into the ear through two-small tubes [30,44,45,46]. The frequencies of two-testing sounds were determined by a geometric mean of *f*_1_ and *f*_2_ (*f*_0_ = (*f*_1_ × *f*_2_)^1/2^) at *f*_0_ = 4, 8, 16, and 20 kHz with *f*_2_/*f*_1_ = 1.22. The intensity of *f*_1_ (*I*_1_) was set at 5 dB SPL higher than that of *f*_2_ (*I*_2_). The distortion product was recorded with an average of 150 times. A cubic distortion product of 2*f*_1_ – *f*_2_ was measured as DPOAE.

### 4.4. Cochlear Preparation and Immunofluorescent Staining

The detailed methods and procedures of immunofluorescent staining can be found in our previous reports [8,41]. After decapitation, the cochlea was isolated and fixed with 4% paraformaldehyde for 0.5–1 h. After decalcification with 10% EDTA for 2 days, the cochlea was embedded with OCT (Cat # 4583, Sakura Finetek USA Inc., Torrance, CA, USA), and cut into 10-µm thick sections at −22~24 °C by a cryostat (Thermo Electron Corp., Waltham, MA, USA). For immunofluorescent staining, the sections were incubated in a blocking solution, which was composed of 10% goat serum and 1% bovine serum albumin, with 0.1% Triton X-100 for 30 min, and then reacted with chicken anti-human Panx1 antibody (1:500; #4515, a gift from Gerhard Dahl at the University of Miami Medical School) and polyclonal rabbit anti-Cx30 antibody (1:400, #71-2200, Invitrogen, Carlsbad, CA, USA) or monoclonal mouse anti-Cx26 (1:400, # 33-5800, Invitrogen, CA) at 4 °C overnight. The specificity of this Panx1 antibody was verified in previous publications by Western blotting and Panx1 KO mouse tissues in our laboratory and other laboratories [8,9,21,30,37,38]. After washout with PBS for three times, the sections were incubated with corresponding secondary antibodies (Alexa Fluor 488- and 568, 1:500, Molecular Probes, Eugene, OR, USA) at room temperature (23 °C) for 2 h to visualize the staining. The staining was observed under a fluorescence microscope (Nikon 2000, Nickon, Melville, NY, USA).

### 4.5. Cochlear Epithelium Whole-Mounting and Hair Cell Loss Accounting

As reported in our previous publications [21,44,45], the cochlear epithelia were isolated and stained with phalloidin-Alexa Fluor-488 and propidium iodide in the whole-mounting preparation. Hair cells were accounted under a 20× lens.

## 5. Conclusions

Our present study demonstrated that Gen-Panx1 KO mice have hearing loss. These data are consistent with previous observations from other Panx1 KO mouse lines [21,30] and the known occurrence of Panx1 mutation-induced hearing loss in humans [31]. These new data further confirm that Panx1 deficiency can induce hearing loss. However, Panx1 function in hearing still remains largely undetermined and needs to be further studied in the future. Also, as demonstrated by this study, multiple auditory function tests, careful characterization of Panx1-deficient mice, and specificity of anti-Panx1 antibody are important and required for assessing the role of Panx1 in hearing and other Panx1 functions. 

## Abbreviations

ABR	auditory brainstem response
CM	cochlear microphonics
cKO	conditional knockout
DPOAE	distortion product otoacoustic emission
EMMA	European Mouse Mutant Archive
EP	endocochlear potential
KO	knockout
OC	organ of Corti
OHC	outer hair cell
OSC	outer sulcus cell
PTS	permanent threshold shift
SLM	spiral limbus
WT	wild-type
